# Applying openEHR’s Guideline Definition Language to the SITS international stroke treatment registry: a European retrospective observational study

**DOI:** 10.1186/s12911-016-0401-5

**Published:** 2017-01-10

**Authors:** Nadim Anani, Michael V. Mazya, Rong Chen, Tiago Prazeres Moreira, Olivier Bill, Niaz Ahmed, Nils Wahlgren, Sabine Koch

**Affiliations:** 1 Health Informatics Centre, LIME, Karolinska Institutet, Tomtebodavägen 18, Stockholm, SE 17177 Sweden; 2 Department of Clinical Neuroscience, Karolinska Institutet, Stockholm, Sweden; 3 Department of Neurology, Karolinska University Hospital Solna, Stockholm, Sweden; 4 Cambio Healthcare Systems, Stockholm, Sweden; 5 Department of Neurology, Centre Hospitalier Universitaire Vaudois (CHUV), Lausanne, Switzerland

**Keywords:** Knowledge management, Standards, Registries, Practice guidelines, Guideline adherence

## Abstract

**Background:**

Interoperability standards intend to standardise health information, clinical practice guidelines intend to standardise care procedures, and patient data registries are vital for monitoring quality of care and for clinical research. This study combines all three: it uses interoperability specifications to model guideline knowledge and applies the result to registry data.

**Methods:**

We applied the openEHR Guideline Definition Language (GDL) to data from 18,400 European patients in the Safe Implementation of Treatments in Stroke (SITS) registry to retrospectively check their compliance with European recommendations for acute stroke treatment.

**Results:**

Comparing compliance rates obtained with GDL to those obtained by conventional statistical data analysis yielded a complete match, suggesting that GDL technology is reliable for guideline compliance checking.

**Conclusions:**

The successful application of a standard guideline formalism to a large patient registry dataset is an important step toward widespread implementation of computer-interpretable guidelines in clinical practice and registry-based research. Application of the methodology gave important results on the evolution of stroke care in Europe, important both for quality of care monitoring and clinical research.

**Electronic supplementary material:**

The online version of this article (doi:10.1186/s12911-016-0401-5) contains supplementary material, which is available to authorized users.

## Background

Several standardisation efforts are being made to tackle the issue of widely non-interoperable health information systems. Reaching interoperability would mean that different health information systems could exchange information between each other and the receiving system would additionally understand what has been sent to it, and not only be able to read it. Examples of those efforts are the standardisation specifications developed by Health Level Seven International (HL7) [1], the International Organization for Standardization (ISO) [2], the openEHR Foundation [3], the Clinical Information Modeling Initiative (CIMI) [4], the International Health Terminology Standards Development Organisation (IHTSDO) [5] and the World Health Organization (WHO) [6].

The means by which these efforts aim to reach interoperability differ in the sense that the proposed solutions address different aspects of interoperability. While some of them, e.g. HL7 FHIR, the ISO standard 13606 and openEHR, offer standardised information models which include data types and classes that are tailored to health care, others focus more on providing standardised terms, e.g. IHTSDO and WHO through their terminologies SNOMED CT and ICD respectively. The different approaches are, however, interconnected as data elements from the former kind can often be bound to standardised terms from the latter type of initiatives.

Clinical practice guidelines (from now on referred to as ‘guidelines’) are ‘systematically developed statements to assist practitioner and patient decisions about appropriate health care for specific circumstances’ based on the latest evidence in the respective clinical field, e.g. diagnostic or treatment-related decisions [7]. Complying with guideline recommendations is expected to increase the numbers of patients treated according to best evidence and improve outcomes.

While guidelines are products of research, registries are tools that store patient data tailored for conducting research on a particular disease or treatment modality. Registries obtain their data in different ways, e.g. directly from electronic health records or through manual data entry by designated health care professionals.

Here we report on the use of a formalism for modelling guideline knowledge based on openEHR specifications, the Guideline Definition Language (GDL), to represent stroke guidelines and run the resulting computer-interpretable guidelines on data from the Safe Implementation of Treatments in Stroke (SITS) registry. GDL is a rule-based language that has recently been added to the openEHR specifications [8]. The SITS registry is a prospective, multinational, observational registry for medical centres documenting stroke treatments since 2002 [9].

We have previously experimentally tested a methodology in which we modelled guideline knowledge using GDL, in order to check the compliance of 49 mock stroke patient cases with European Stroke Organisation guidelines, with promising results [10]. The implications of the success of such a methodology are obtaining shareable evidence-based knowledge models that can be used not only for guideline compliance checking but also be deployed (together with workflow, user interface and other components) in clinical decision support systems assisting practitioners and patients at the point of care.

In order to further validate our methodology, the aim of this project was to test it on thousands of real stroke patient case files from the SITS registry and compare the results to those obtained from conventional data analysis using standard statistical software, which we recently reported for a clinical audience [11].

## Methods

**Fig. 1:**
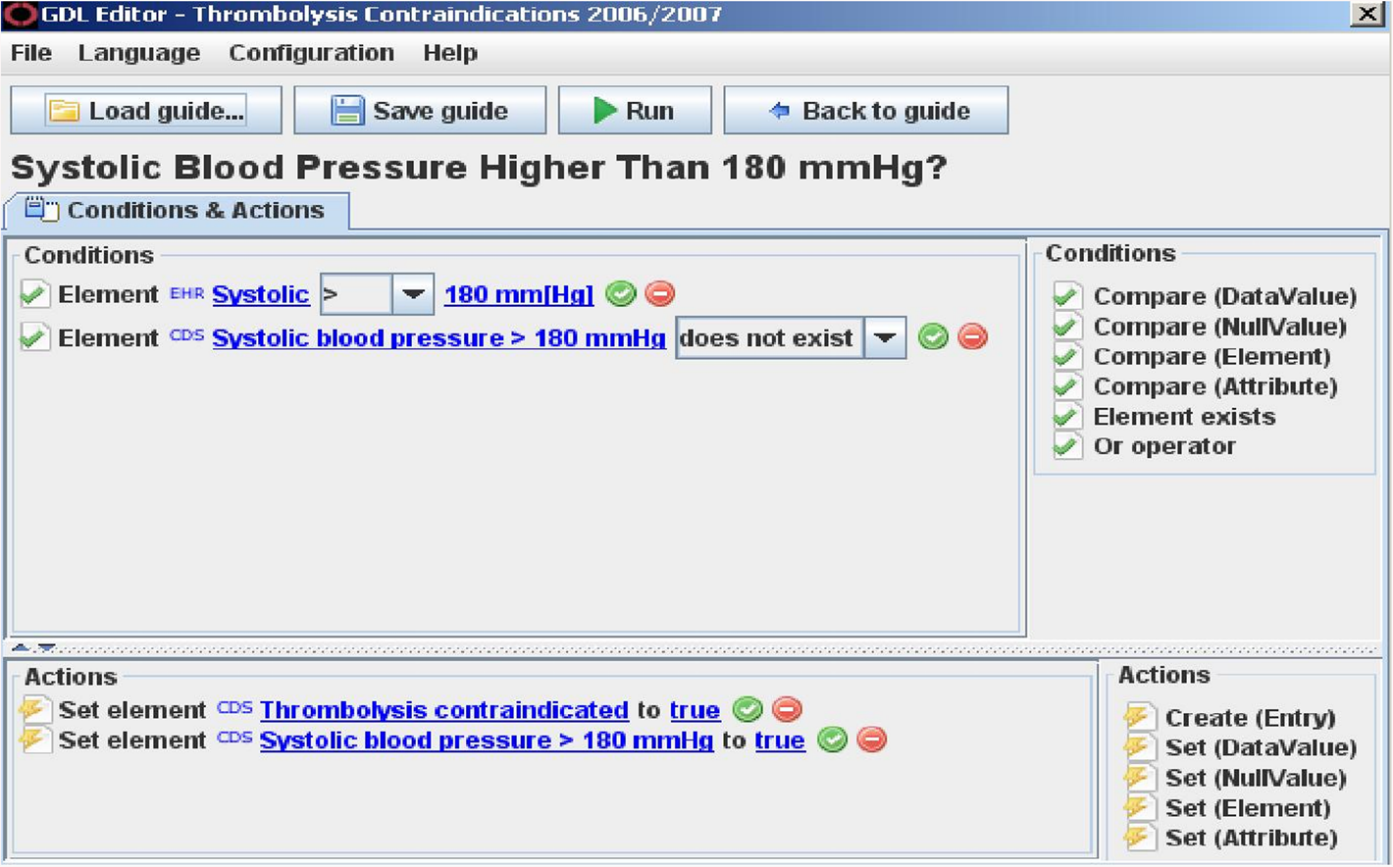
A GDL Rule. A GDL rule as presented within the free and open-sourced tool GDL Editor. Here exceeding a blood pressure threshold contraindicates thrombolysis treatment. The blue parts constitute data elements coming from reusable clinical content models in the form of archetypes

We have described the openEHR-based methodology previously, when tested in an experimental setting [10]. The following summarises the main elements of this methodology:

- Archetypes: reusable models of clinical concepts, which undergo review in order to standardise them. An archetype could, for example, represent a blood pressure observation or diagnosis decision. Archetypes can be reviewed on regional, national and international levels. They are supposed to represent maximal data sets, i.e. if an archetype represents a diagnosis decision, then any organisation needing to deal with diagnosis-related data should be able to retrieve the data it needs from a high quality diagnosis archetype. Also, archetype data elements can be bound to standardised terms from terminologies like SNOMED CT or ICD.
- GDL rules: rules defined in GDL base their data acquisition on archetype data elements as well as provide their output (an alert, for instance) as archetype data elements. GDL, too, supports binding data to terminologies. Data in GDL rules can be associated with multiple terminologies simultaneously. Figure 1 shows an example of a GDL rule.

It is worth noting that our methodology does not support workflow aspects, adaptive behaviour or the specification of clinical intentions and exceptions. Within a clinical decision support system, these components and other process modelling technologies would complement GDL technology in its current form.

The tools we used to apply our methodology are:

- The international archetype repository,^1^ i.e. the international instance of the ‘Clinical Knowledge Manager’, to retrieve existing archetypes.
- The free and open-sourced Archetype Editor^2^ to author new archetypes or modify existing ones.
- The free and open-sourced GDL Editor^3^ to author GDL rules.
- The CDS Workbench by Cambio Healthcare Systems to run GDL rules on archetyped data.

The details of the produced archetypes and GDL rules, which have not changed for this use case, can be found in our previous publication [10]. For mapping data from the SITS registry into a format that the CDS Workbench can work with and running the compliance checks, we went through the following steps:

1. SITS registry data, in form of a comma-separated values (CSV) file, were split into multiple CSV files which aligned with archetype structures. For each archetype, e.g. a blood glucose archetype, we created a separate CSV file that contained the data related to that particular medical aspect of European stroke treatment recommendations (blood glucose thresholds, in this case), in addition to patient treatment file numbers (see Additional files 1, 2, 3, 4, 5 and 6 for examples of the produced files).
  In our use case of acute stroke treatment, the mapping was rather straightforward. Data elements from the original CSV files were either available in existing archetypes or we created archetypes for them where we could not find those data elements in common repositories (without creating redundancy or overlap in archetype concepts between new and existing archetypes). We did not encounter the problem of using an archetype with mandatory data values that were not available in the registry. In the opposite case, it was possible to modify an archetype by extending its data definitions.
  We decided whether a registry data item was equivalent to an archetype data element by
  discussing the meaning of the registry data item with SITS clinical specialists if the data item’s label left any reason for doubt,
  checking the description of the archetype data element through the description part that accompanies every archetype data element and checking the general archetype meta-data (in addition to archetype review information, if necessary) and then
  judging the equivalence.
2. We ran GDL rules on the CSV files using the CDS Workbench. The CDS Workbench essentially connects the relevant GDL rules with archetyped patient data. The CDS Workbench operates directly on the newly created CSV files, creating in-memory representations of the relevant archetype objects and GDL rules. Note also that the GDL rules as such are specified in terms of archetype data elements, e.g. in the rule
["gt0004"] = (RULE) <
when = <"$gt0020==null",
"($gt0018<50,mg/dl)||($gt0018>400,mg/dl)">
then = <"$gt0049=true", "$gt0020=true">
  the code gt0018 represents an archetype element for blood glucose as a quantity and is directly derived from a corresponding archetype in a previous section of the GDL code:
["gt0017"] = (ARCHETYPE_BINDING) <
archetype_id = <"openEHR-EHR-OBSERVATION.lab_test-
blood_glucose.v1">
domain = <"EHR">
elements = <
["gt0018"] = (ELEMENT_BINDING) <
path =
<"/data[at0001]/events[at0002]/data[at0003]
/items[at0078.2]"
3. The CDS Workbench produced proportions of patients treated in non-compliance with guidelines.
4. We created additional GDL rules to account for missing values, which the CDS Workbench then counted so that we could exclude them from the compliance calculations. This was necessary since the standardised archetype objects were in-memory, without the ability to affect them directly in the compliance checking configuration (see also point 2 above).

We applied this method to check the compliance of patient cases (18,400 patients) from across Europe (19 countries, 232 hospitals) with European Stroke Organisation guidelines and European Medicines Agency regulations for using intravenous thrombolysis in the treatment of acute ischaemic stroke. We have reported the criteria that led to our patient samples and their clinical importance, which was to provide a basis for studying the way compliance with recommendations changed following a guideline update. The update had been introduced in two documents published in 2008 and 2009, so we studied two samples of patients from hospitals enrolling cases in SITS both in the years 2006 to 2007 (*n* = 6354) as well as 2010 to 2011 (*n* = 12,046) [11].

Finally, we compared the compliance rates from our method presented here with the results we obtained in the clinical study above [11], which had used conventional statistical analysis.

## Results

**Fig. 2:**
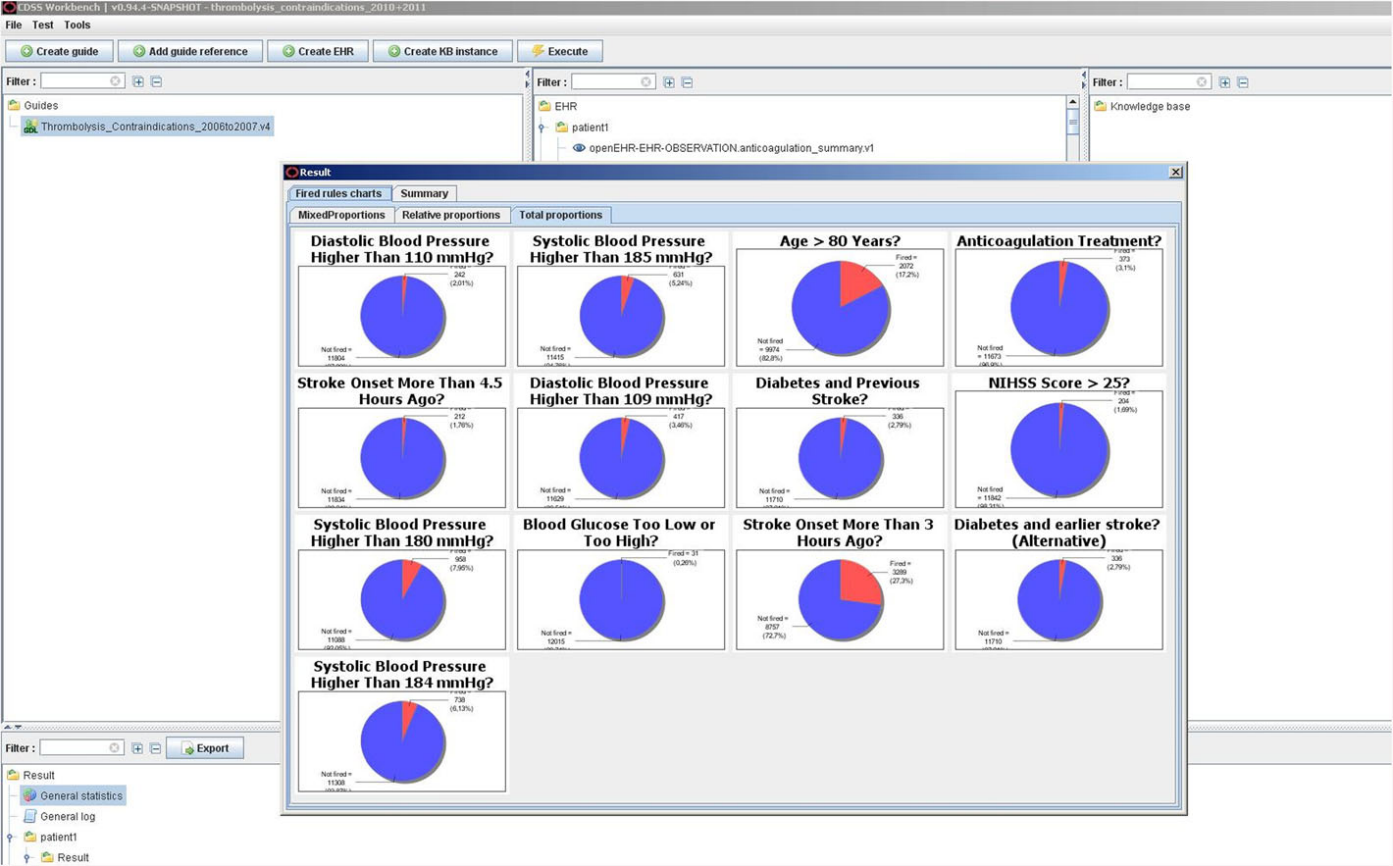
Results Display. The tool CDS Workbench produced non-compliance rates as pie charts using GDL. Each pie chart relates to one of the contraindications to thrombolysis treatment in acute ischaemic stroke as laid out in European guidelines and regulations

Our method yielded the same rates of non-compliance as using conventional statistical software. This shows that GDL-based technology is reliable when applied to thousands of real patient data files (from 18,400 patients in this case) and thus applicable to large and complex clinical registries. Table 1 and Fig. 2 show examples of our results.

**Table 1:** Results of non-compliance obtained by openEHR’s Guideline Definition Language (GDL) and compared to conventional analysis

Contraindication to Thrombolysis Treatment	Non-Compliance (%)			
	2006–2007		2010–2011	
	CA	GDL	CA	GDL
NIHSS score > 25	1.1	1.1	1.7	1.7
Anticoagulation treatment	2.4	2.4	3.1	3.1
Systolic blood pressure > 185 mmHg	2.5	2.5	5.4	5.4
Systolic blood pressure > 184 mmHg	4.0	4.0	6.3	6.3
Diastolic blood pressure > 110 mmHg	0.8	0.8	2.1	2.1
Diastolic blood pressure > 109 mmHg	2.1	2.1	3.6	3.6
Diabetes and previous stroke	2.5	2.5	2.8	2.8
Blood glucose < 50 mg/dl or > 400 mg/dl	0.3	0.3	0.3	0.3
Age > 80 years	8.9	8.9	17.2	17.2
Stroke onset > 3 h ago	8.2	8.2	27.9	27.9
Stroke onset > 4.5 h ago	0.9	0.9	1.8	1.8

The guidelines and regulations from the European Stroke Organisation [23, 24] and European Medicines Agency [25] respectively are in the form of contraindications to the use of a decisive stroke treatment – intravenous thrombolysis. These results from GDL and archetypes completely match results from conventional statistical software where the clinical question was studied in detail [11]

*CA* conventional analysis, *NIHSS* National Institutes of Health Stroke Scale

This also shows that our methodology can be used to assist in monitoring of quality of care and in clinical research. In this case, the GDL technology showed that clinicians promptly adhered to the European guideline update from 2009 that increased the time window for stroke treatment from 3 to 4.5 h after symptom onset, reflected by higher non-compliance with the contraindication ‘Stroke onset > 3 h ago’ in 2010 and 2011. Similarly, the guideline update allowed the treatment of patients aged over 80 years, also reflected by higher non-compliance with the contraindication ‘Age > 80 years’ (Table 1).

The results obtained here were a complete match for each single patient, regarding all compliance criteria. Since there were no exceptions to this, presenting our results on a case-by-case (patient-by-patient) basis would not contribute additional insight, as all compliance criteria we checked (see Table 1) evaluated to either true or false.

## Discussion

We have demonstrated for the first time that it is possible to retrospectively check the compliance of thousands of real patient cases with guideline recommendations using a technology that is based on openEHR’s Guideline Definition Language and a patient data registry. We utilised the SITS registry for obtaining patient data. By doing so, we validated the results of a previous experiment that was limited to a small number of mock patient cases [10], and took an important step toward further implementation of such methods in electronic health record systems and patient registries.

### Limitations

One limitation of this study is related to tools supporting the use of GDL. The CDS Workbench did not have built-in functions to deal with missing data values, so we had to create extra GDL rules in the GDL Editor. Furthermore, we had to map the SITS registry database to an openEHR-specific CSV database, which constituted an additional work step.

The fact that GDL artefacts provided both guideline recommendations and a means of configuring the compliance checking implementation (e.g. to deal with missing values) presents a problem of different purposes being mixed together, and may thus make GDL rules less maintainable and less reusable in the long term.

Another limitation is that this technology is not suited to measure the statistical significance of the obtained results. Such calculations would typically have to be done by a separate application.

### Mapping vs. interoperability

Our methodology could not be applied directly to the CSV file that was exported from the SITS registry and thus this work included a manual mapping step (see the section Limitations above), which can be seen to hamper interoperability. The target of the mapping still followed the structure of standardised archetypes, producing shareable mapped artefacts if another system wants to implement the same kind of openEHR-specific CSV database. Optimally, an openEHR-based system would provide the underlying structure of the registry in question.

Also, when dealing with legacy systems, some form of manual mapping will always be required if the original model is not used to represent clinical guidelines.

### The nature of clinical practice guidelines and how GDL fits in

In our experience, clinical practice guidelines from different clinical domains (i.e. not only from acute stroke management) tend to contain their recommendations in a condensed form that is transferrable to computational formats of the *if-then-else* type. Furthermore, it is becoming increasingly common to release guidelines with accompanying flowcharts (e.g. guidelines released by NICE [12] and NGC [13]) that are equally computable with rule-conformant structures as they take place in the GDL implementation presented herein.

However, it is important to keep in mind that there are still certain clinical scenarios (e.g. complex chemotherapy regimens) that require more complex structures than GDL functionality can accommodate at this point. A complete clinical situation with all its socio-technical complexities also requires additions to pure rule-based logic such as workflow support and situation awareness.

Having said that, it is also important to be aware of the characteristics of a guideline’s recommendations when attempting to computerise those using GDL. While GDL’s functionality has been undergoing continuous improvements since the release of the language in 2013, it is helpful to keep in mind that the typical use cases today are those that can be represented using common mathematical functions that entail comparison of data values to each other or thresholds using mathematical operators, or involve setting values using standardised terminology codes or mathematical formulae.

Arguably one of the biggest strengths of GDL lies in its ability to solve the ‘curly braces problem’, i.e. the inefficient reliance on locally defined data models for retrieving data needed by guideline logic. GDL solves that by being based on archetypes, standard data models and standard terminologies. Furthermore, technical concerns such as runtime rules’ language syntax and mappings to local clinical models are not mixed with the clinical views of the rules which are meant to be used and verified by clinicians.

### Implications for patient care

Being able to check guideline or protocol compliance on retrospective patient data can give managers or other stakeholders in health care an opportunity to see the performance of their respective organisations when it comes to following the latest evidence and state of the art in their fields. Also, it allows conducting clinical research that may investigate reasons for certain compliance patterns.

### Interoperability, shareability and reusability

When the above benefits are based on interoperability specifications this adds the advantages that

- data from several organisations, which follow a certain interoperability standard, can be investigated at the same time and
- all organisations implementing a certain interoperability standard can share and maintain the same guideline knowledge models.

The execution of standardised models of clinical and guideline knowledge, like openEHR archetypes and GDL rules, does not have to be limited to retrospective checking of guideline compliance, but could enhance clinical decision making at the point of care. The same GDL rules used for compliance checking could, for instance, lead to notifying a clinician about a deviation from a guideline recommendation in a prescription order they have just placed.

### openEHR-based clinical decision support

While this work focussed on retrospective functionality, there have long been aspirations and experiences when it comes to using openEHR technologies for providing point-of-care decision support.

González-Ferrer et al. report positive results about the adequacy of openEHR archetypes within the specification of decision support-specific clinical statements [14]. Chen et al. demonstrate initial successes in deploying a regional clinical decision support system based on GDL [15]. In a further effort, Xiao et al. use archetypes to develop decision support functionality in the clinical area of methadone-based therapy [16].

Meanwhile, when looking at a higher level picture of how different semantic components come together to achieve clinical decision support, the work herein fits into three of the layers recently published by the European SemanticHealthNet project: the openEHR archetypes fit into the layer of structured heterogeneous data (layer 1), and the GDL rules fit into the layers of semantic mediators (layer 3) and virtual homogeneous data (layer 4). Reaching a full clinical decision support system with sound semantics will entail addressing the remaining two layers, but some of that work falls outside the scope of interoperability specifications as such, e.g. when application developers realise the layer of applications based on their particular business use cases [17].

### Implications for the development and maintainability of health records

In the long run, reusability, shareability and interoperability advantages are likely to materialise into economic advantages when developing and maintaining electronic health records as well. Having reusable clinical content models in the form of archetypes and reusable guideline knowledge models in the form of GDL rules will further foster effective software development and maintainability. At the same time, the separation of clinical models from technical models (two-level modelling), which is one of the core openEHR solution constituents, will further foster effective software engineering by harnessing the benefits of model-driven development. Studies into this have varied in their findings, where some had positive insights about the effects of model-driven development in openEHR-based solutions [18] and others discovered challenges that still need to be solved in real world implementations [19].

### Directions for future work

Evaluating GDL technology at the point of care is an important next step, as it could also lead to a better understanding about features which may be missing from GDL’s functionality.

Also, introducing measures and procedures to achieve quality assurance of GDL rules that may interest a wider community could prove useful. One example of doing this would be reviewing GDL rules the way archetypes are reviewed today, which could lead to the advantage of clear categorisation of GDL rules into the clinical domains they belong to. Additionally, modifying GDL to address a better separation of concerns where GDL rules strictly deal with clinical guideline content and nothing else would also lead to GDL rules of higher shareability and maintainability value.

Furthermore, more comparisons are needed with similar research that has used other interoperability standards than openEHR or other computer-interpretable guideline formalisms than GDL such as PROforma [20], Asbru [21] or SAGE [22]. Eventually, it would be desirable for more and more agreement to take place regarding a minimum set of necessary functions for computerising clinical practice guidelines for different purposes, which, in turn, could lead to more standardisation and potentially better patient care.

## Conclusions

Registry-based analyses of compliance with clinical practice guidelines are possible with state-of-the-art technologies for interoperability. openEHR archetypes and GDL rules could be applied to detect deviations from best practice, guidelines or regulations. Furthermore, such technologies may be worth considering when taking measures to improve continuity of care, quality of care, clinical research and clinical decision making.

## Additional files

Additional file 1: Patient Ages and Times Since Stroke Onset – contains data on patient age and time since stroke onset for the 12,046 patients of the 2010–2011 study period. (CSV 315 kb)
Additional file 2: Previous Strokes and Diabetes – contains data on previous stroke and diabetes diagnoses among the 12,046 patients of the 2010–2011 study period. (CSV 426 kb)
Additional file 3: NIHSS Scores – contains data on the NIHSS scores of the 12,046 patients of the 2010–2011 study period. (CSV 183 kb)
Additional file 4: Anticoagulation Treatments – contains data on anticoagulation therapy for the 12,046 patients of the 2010–2011 study period. (CSV 223 kb)
Additional file 5: Blood Pressures – contains data on blood pressure values of the 12,046 patients of the 2010–2011 study period. (CSV 396 kb)
Additional file 6: Blood Glucose Measurements – contains data on blood glucose values of the 12,046 patients of the 2010–2011 study period. (CSV 271 kb)

## Abbreviations

GDL: Guideline Definition Language
guidelines: clinical practice guidelines
SITS: Safe Implementation of Treatments in Stroke

## Footnotes

1
http://www.openehr.org/ckm

2
http://www.openehr.org/downloads/archetypeeditor/home

3
http://sourceforge.net/projects/gdl-editor/
